# Management of femoral head fracture by Ganz surgical dislocation of the hip

**DOI:** 10.1186/s10195-022-00643-w

**Published:** 2022-05-10

**Authors:** Hossam Hosny, Shazly Mousa, Wael Salama

**Affiliations:** Orthopedic and Traumatology Department, Faculty of Medicine, Sohag University, Sohag, Egypt

**Keywords:** Hip dislocation, Femoral head fracture, Pipkin classification, Ganz technique, Femoral head osteonecrosis, Trochanteric flip osteotomy

## Abstract

**Introduction:**

Posterior hip dislocation is the commonest type of hip dislocation. It is associated with femoral head fracture in 7% of cases. Urgent and congruent hip reduction is mandatory to improve clinical outcomes and avoid irreversible complications. The purpose of this study is to assess the safety and functional and radiological outcomes of surgical hip dislocation by Ganz technique for treatment of femoral head fracture.

**Patients and methods:**

In this retrospective study, 18 cases of femoral head fracture were included. Six cases had Pipkin type I and 12 had Pipkin type II fracture. They were treated through surgical hip dislocation. All cases were followed up for at least 24 months. Matta’s criteria were used for radiological evaluation (plain radiographs). Functional evaluation was done using Harris Hip Score and modified Merle d’Aubigne and Postel score at final follow-up.

**Results:**

No patients were lost during the follow-up period. No signs of infection or wound dehiscence were noted in this study. There was one case of osteonecrosis. All cases had labral injury, which was debrided. None of our cases needed suture anchor repair of the labrum. Radiographical evaluation according to Matta’s criteria yielded anatomic fracture reduction in 17 patients but imperfect in 1 patient. According to Harris Hip Score, four Pipkin type I cases were rated as excellent and two as good. Among cases of Pipkin type II fracture, six were rated as excellent, four as good, one as fair, and one as poor. According to modified Merle d’Aubigne and Postel score, 11 cases had excellent results, 5 cases were rated as good, one as fair, while one case had poor results.

**Conclusion:**

Open reduction and internal fixation of femoral head fracture using surgical hip dislocation through Ganz approach is a viable treatment option and provides satisfactory results with low complication rate.

## Introduction

Femoral head fracture is considered to be an uncommon injury that occurs as a result of high-energy trauma and usually results from traumatic hip dislocation. Delayed diagnosis inevitably leads to poor prognosis and irreversible complications that may eventually require hip joint arthroplasty [1]. Because of the infrequency of femoral head fracture, few studies are available, and often with only a small number of patients [1–8]. The association of femoral head fracture and hip dislocation was first described in 1869 by Birkett [9]. In 1957, Pipkin published a new classification system for femoral head fracture [10]. Several surgical approaches have been described for treatment of femoral head fracture. These approaches have been associated with a high complication rate [2, 4, 5, 11, 12]. They do not provide full view of the femoral head. Surgical hip dislocation provides this advantage [4, 13]. It preserves and protects the blood supply of the femoral head and allows full access to the femoral head [2, 4, 13]. Reinhold Ganz described a surgical hip dislocation technique and reported no osteonecrosis in his case series [13]. Few literature studies describe the technique of surgical hip dislocation [1, 2, 4–6, 8, 14]. The purpose of this study is to assess the safety and functional and radiological outcomes of surgical hip dislocation by Ganz technique for treatment of femoral head fracture.

## Patients and methods

This monocentric retrospective study was performed in a tertiary referral center and considered all patients treated consecutively from 2014 to 2018 using surgical hip dislocation technique. Eighteen cases with posterior hip dislocation associated with femoral head fracture were included. All procedures were done by three fellowship-trained orthopedic surgeons. Average time to operation was 5 days (range 3–7 days). The patients and their relatives were informed about the surgical technique and its complications. They should know that negative drilling test carries a very high risk but is not consistent of osteonecrosis. Osteonecrosis can still occur even if the drilling test is positive. Drilling test is intraoperative drilling of the femoral head with a Kirschner wire to assess the femoral head viability. The study was approved by the local Ethics Committee of our institution.

### Inclusion criteria

We included all patients with all types of Pipkin fracture requiring internal fixation (Pipkin type I and type II with displaced femoral head fractures more than 2 mm).

### Exclusion criteria

1. Neglected cases (hip dislocation neglected for more than 24 h)
2. Osteoarthritic and dysplastic hips
3. Follow-up of less than 2 years

## Methods

All patients were admitted at the emergency department. The advanced trauma life support guidelines were followed in the resuscitation protocol. Once the patients were hemodynamically stable, routine plain radiographs of the pelvis (anteroposterior view, obturator view, and iliac view) were performed. If hip dislocation was diagnosed on plain radiograph, closed reduction was done under anesthesia. Three-dimensional computed tomography was requested for cases with femoral head fracture after hip reduction. Irreducible hips were amenable to open reduction under general anesthesia. Skin traction or skeletal traction was applied after hip reduction.

### Surgical technique

All patients were placed in lateral decubitus position under spinal anesthesia. After routine antiseptic measures and lower limb preparation, a direct lateral approach was used in all cases. A 15-cm incision was centered on the greater trochanter. Dissection extended proximally to overlying gluteus maximus fascia and distally to the fascia lata layer. Then, the fascia lata layer was incised longitudinally and proximally from the most distal extent of the wound up to the greater trochanter. The incision was continued along the anterior border of the gluteus maximus, which was relaxed by hip extension and knee flexion. This allowed more exposure of the posterior border of the gluteus medius.

Once the fascia was completely opened, the following landmarks were readily identifiable: greater trochanter, gluteus medius, and vastus lateralis muscles. At this stage, the trochanteric bursa and the loose areolar tissue overlying the short external rotators were still intact. It is recommended that a flap of trochanteric bursa and the attached tissues are prepared with an anterior incision and retracted posteriorly. This flap was repositioned and repaired when the wound was closed, preventing adhesions between the greater trochanter and the fascia lata.

Once the piriformis tendon was visible, we identified the interval between the piriformis tendon and the posterior border of the gluteus minimus. This interval was sharply dissected over a distance of 1–2 cm. The osteotomy was started from the posterosuperior border of the greater trochanter and extended to the border of the vastus lateralis muscle. The trochanter was cut with an electric saw in a posteroanterior direction. The trochanteric fragment should be nearly 1.5 cm thick (a thinner fragment can break easily and is difficult to reattach; a thicker fragment may injure the insertion of external rotators, leading to vascular damage).

The external rotator muscles must be preserved and their insertion into the greater trochanter left undisturbed. Protection of the medial femoral circumflex artery that is located distal to the obturator externus muscle is essential. The trochanter was moved in continuity with anterior soft tissue structures. The remaining anterior periosteum and the remaining part of the gluteus medius were cut using an inside-out technique. The gluteus minimus was dissected from the capsule sharply. The hip was then flexed to relax the anterior structures, and the capsulotomy step was started. A longitudinal incision was made from the trochanter at the anterior edge of the trochanteric osteotomy towards the acetabulum. The second cut runs along the distal anterior insertion of the capsule around the calcar. The third cut runs parallel to the edge of the acetabulum in a posterior direction. If the capsule had a traumatic tear, the tear was carefully incorporated within the capsulotomy.

After opening the capsule, complete dislocation was obtained through continuous traction with a flexed knee, progressive hip flexion, and external rotation of the femur, causing more subluxation and finally dislocation. A bone hook placed around the calcar can be used to dislocate the head. If the ligamentum teres was not torn, it was cut with a long sharp curved scissor with great care to protect the medial reticulum (Weitbrecht’s ligament) which attached to inferior fragment to reduce the risk of osteonecrosis. Visualization, reduction, and definitive fixation of the fracture can be difficult with an intact ligamentum teres. For this reason, the authors routinely cut an intact ligamentum teres as it does not increase the risk of femoral head osteonecrosis if cut.

After dislocation, acetabular cartilage, femoral head cartilage, and labrum were carefully inspected and probed if needed. Small labral tears were debrided, while large labral tears would be sutured with anchors.

**Fig. 1:**
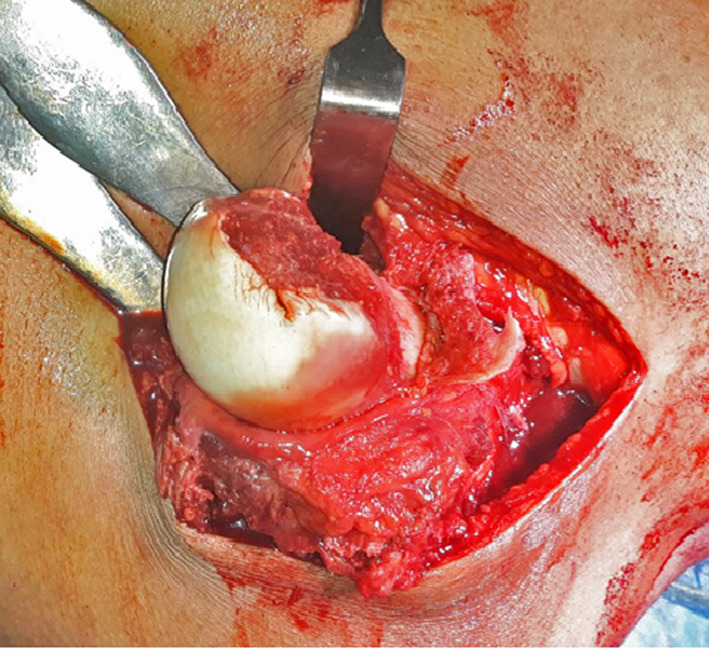
Intraoperative photo showing head of femur with type 1 Pipkin fracture

**Fig. 2:**
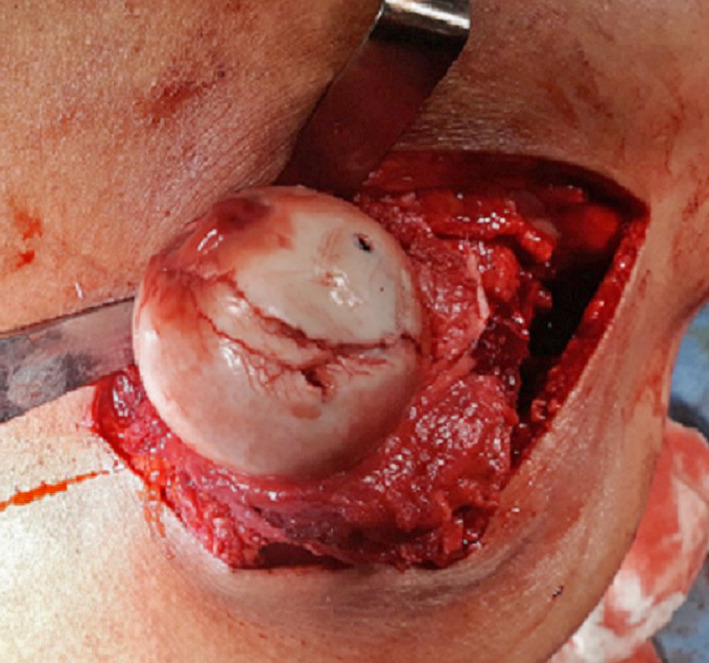
Intraoperative photo showing fixation of head of femur with screws

**Fig. 3:**
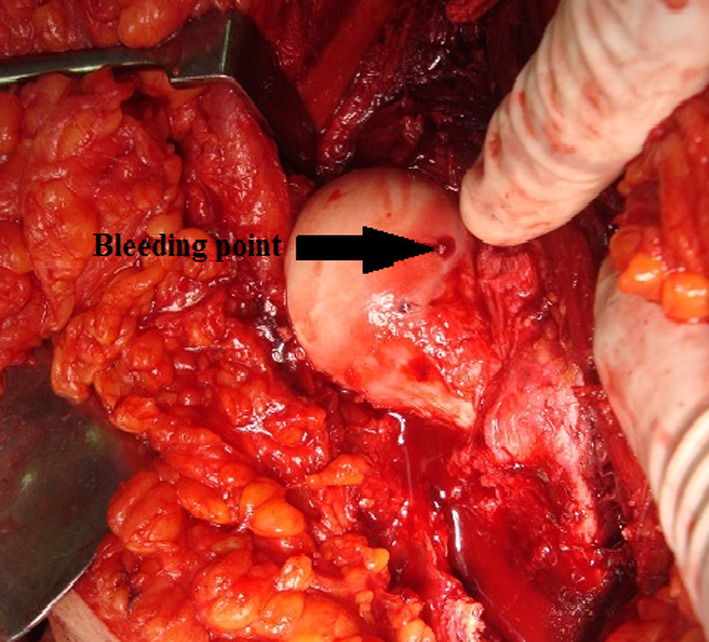
Intraoperative photo showing positive drilling test (arrow)

The fractured fragments of the femoral head were evaluated. Small comminuted fragments (that cannot be fixed) were debrided (never leaving them, as this would result in a loose intraarticular body), while large fragments were reduced anatomically and temporarily fixed with Kirschner wires then fixated using subchondral headless Herbert screws or cannulated screws of 4 mm. Small fragments can be used for head grafting if necessary (Fig. 1, 2). The decision to fix or debride depends on the capability of the fragment to withstand screw fixation even if small fragment. The vascularity of the femoral head was checked through puncture by 1.8-mm K-wire (drilling test) (Fig. 3). Reduction of the femoral head was done. The capsule was repaired without tension to avoid injury of the retinacular vessels. The greater trochanter flap was reduced and fixed with either 6.5-mm cancellous screws or cortical screws of 4.5 mm.

### Postoperative protocol

Prophylaxis against thromboembolism (low-molecular-weight heparin) and heterotopic ossification (indomethacin 75–150 mg/day) was started immediately after the operation. Partial weight-bearing with crutches and physiotherapy was allowed after 6 weeks. Full weight-bearing was started at 2 months according to the follow-up. Radiographic evaluation (anteroposterior and lateral views) was carried out at 4, 6, and 12 weeks for the first 2 months then at 6, 9, and 12 months postoperatively to evaluate healing of the femoral head fracture and osteotomy site, implant failure, and development of osteonecrosis.

### Assessment

All cases were subjected to radiological and functional evaluation. The first author was responsible for assessment of patients. The radiological evaluation used Matta’s criteria of reduction to assess the accuracy of reduction and the presence of residual displacement on plain radiographs [anteroposterior and lateral views (axial cross-table view)] [4, 15]. The functional recovery was evaluated according to both the modified Harris Hip Score (HHS) [16] and the modified Merle d’Aubigne and Postel score [17, 18]. Osteoarthritis was evaluated and graded according to the Tönnis classification [19]. Heterotopic ossification was recorded and graded according to the Brooker classification [20]. Conventional radiological parameters were used to assess healing process, viz. bridging of the fracture by bone, callus, bridging of the fracture at three of four cortices, and obliteration of the fracture line and cortical continuity [21].

## Results

Closed reduction of hip dislocation was done for all cases within the first 6 h. The patient was male in 12 cases, while 10 patients had right femoral head fracture. Mean age was 32 years (range 20–50 years). Mean follow-up period was 40 months (range 25–48 months). The mode of trauma was motor car accident (13 cases), motorcycle accident (3 cases), and fall from height (2 cases). Six cases had type I Pipkin fracture, and 12 cases had type II Pipkin fracture. Associated injuries were present in five patients: one case with bilateral tibial fracture, one case with ipsilateral tibial fracture, two cases with head injury, and one case with rib fracture and ipsilateral forearm fracture (Table 1).

**Table 1:** Demographic data of our cases

Case	Age	Sex	Mode of trauma	Affected side	Type of fracture	Type of fixation	Follow-up period (months)	Harris Hip Score	Modified Merle d’Aubigne and Postel Score	Complications
Case 1	30 years	Male	Motor car accident	Right side	Pipkin type II	Herbert screw	48	95 Excellent	Excellent	–
Case 2	20 years	Male	Motor car accident	Right side	Pipkin type II	Cannulated screw 4 mm	45	95 Excellent	Excellent	–
Case 3	35 years	Female	Falling from height	Left side	Pipkin type I	Cannulated screw 4 mm	46	100 Excellent	Excellent	–
Case 4	42 years	Female	Motor car accident	Right side	Pipkin type II	Cannulated screw 4 mm	48	85 Good	Good	–
Case 5	30 years	Male	Motor car accident	Left side	Pipkin type II	Cannulated screw 4 mm	38	75 Fair	Fair	–
Case 6	35 years	Male	Motorcycle accident	Left side	Pipkin type I	Cannulated screw 4 mm	40	100 Excellent	Excellent	–
Case 7	26 years	Male	Motor car accident	Right side	Pipkin type I	Cannulated screw 4 mm	48	95 Excellent	Excellent	–
Case 8	25 years	Male	Motorcycle accident	Left side	Pipkin type II	Herbert screw	45	90 Excellent	Excellent	–
Case 9	28 years	Male	Motorcycle accident	Left side	Pipkin type II	Cannulated screw 4 mm	48	100 Excellent	Excellent	–
Case 10	25 years	Female	Motor car accident	Right side	Pipkin type II	Herbert screw	45	85 Good	Good	–
Case 11	50 years	Female	Motor car accident	Right side	Pipkin type I	Cannulated screw 4 mm	40	85 Good	Good	–
Case 12	28 years	Male	Motor car accident	Right side	Pipkin type II	Cannulated screw 4 mm	25	60 Poor	Poor	Osteonecrosis
Case 13	44 years	Female	Motor car accident	Left side	Pipkin type II	Herbert screw	30	85 Good	Good	–
Case 14	26 years	Male	Motor car accident	Right side	Pipkin type I	Herbert screw	36	100 Excellent	Excellent	–
Case 15	32 years	Male	Motor car accident	Left side	Pipkin type II	Cannulated screw 4 mm	48	85 Good	Good	–
Case 16	40 years	Female	Fall from height	Left side	Pipkin type II	Cannulated screw 4 mm	36	90 Excellent	Excellent	–
Case 17	36 years	Male	Motor car accident	Right side	Pipkin type II	Cannulated screw 4 mm	24	100 Excellent	Excellent	–
Case 18	24 years	Male	Motor car accident	Right side	Pipkin type I	Herbert screw	45	85 Good	Excellent	–

**Fig. 4:**
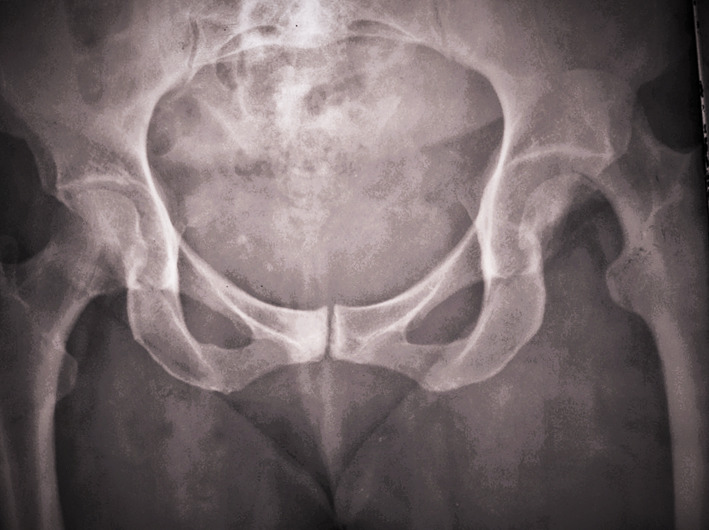
Preoperative radiograph of a female 35-year-old patient with left hip dislocation and fracture of the femoral head (Pipkin type 1 fracture)

**Fig. 5:**
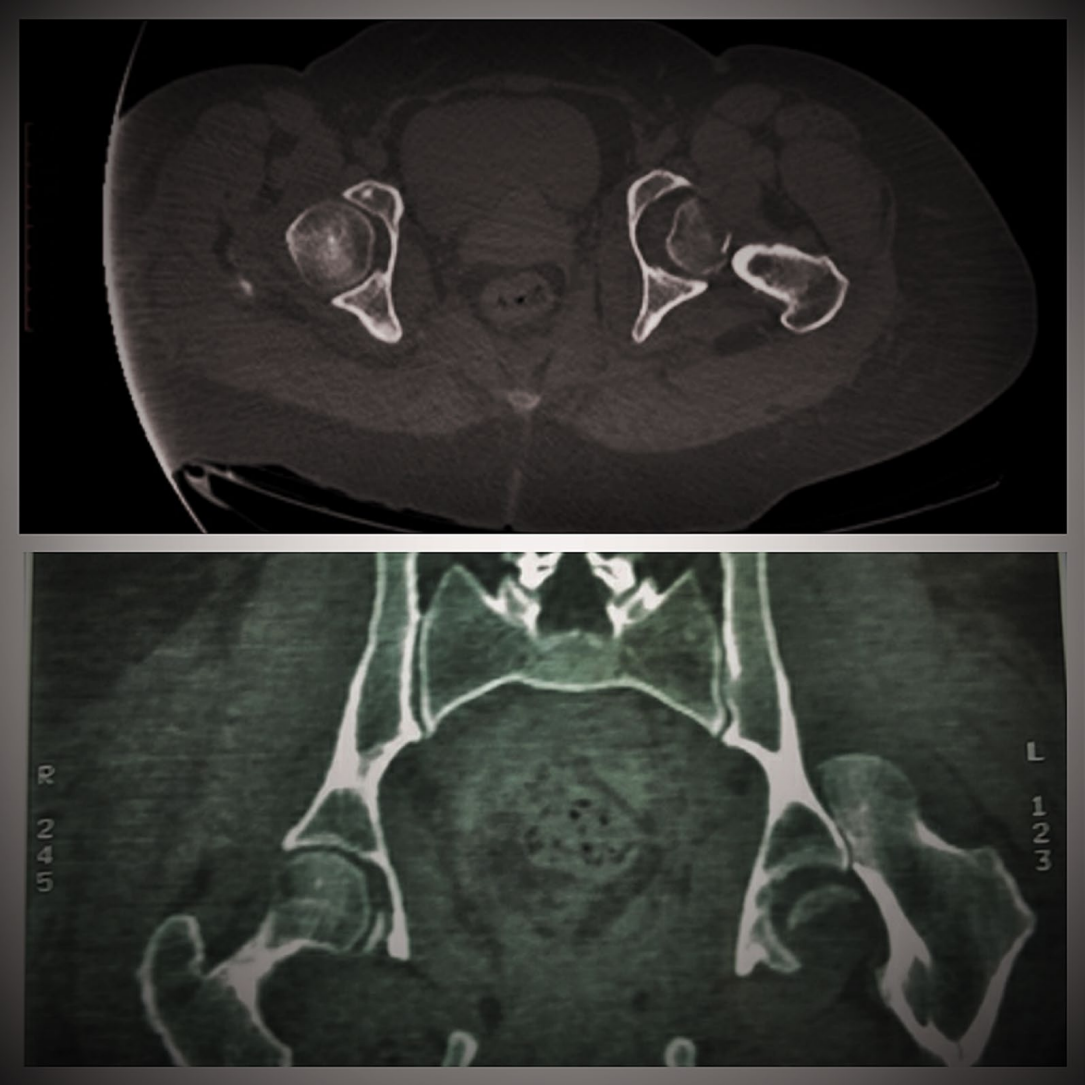
**A** Preoperative CT scan showing head femur fracture (axial view). **B** Preoperative CT scan showing head femur fracture (coronal view)

**Fig. 6:**
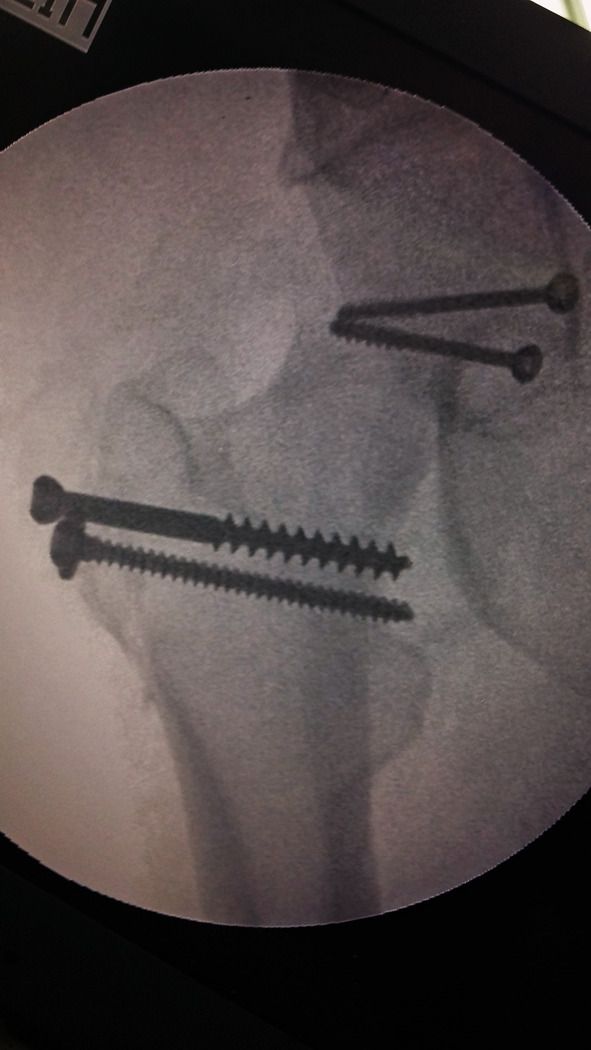
Immediate postoperative plain radiograph showing femoral head fixation

**Fig. 7:**
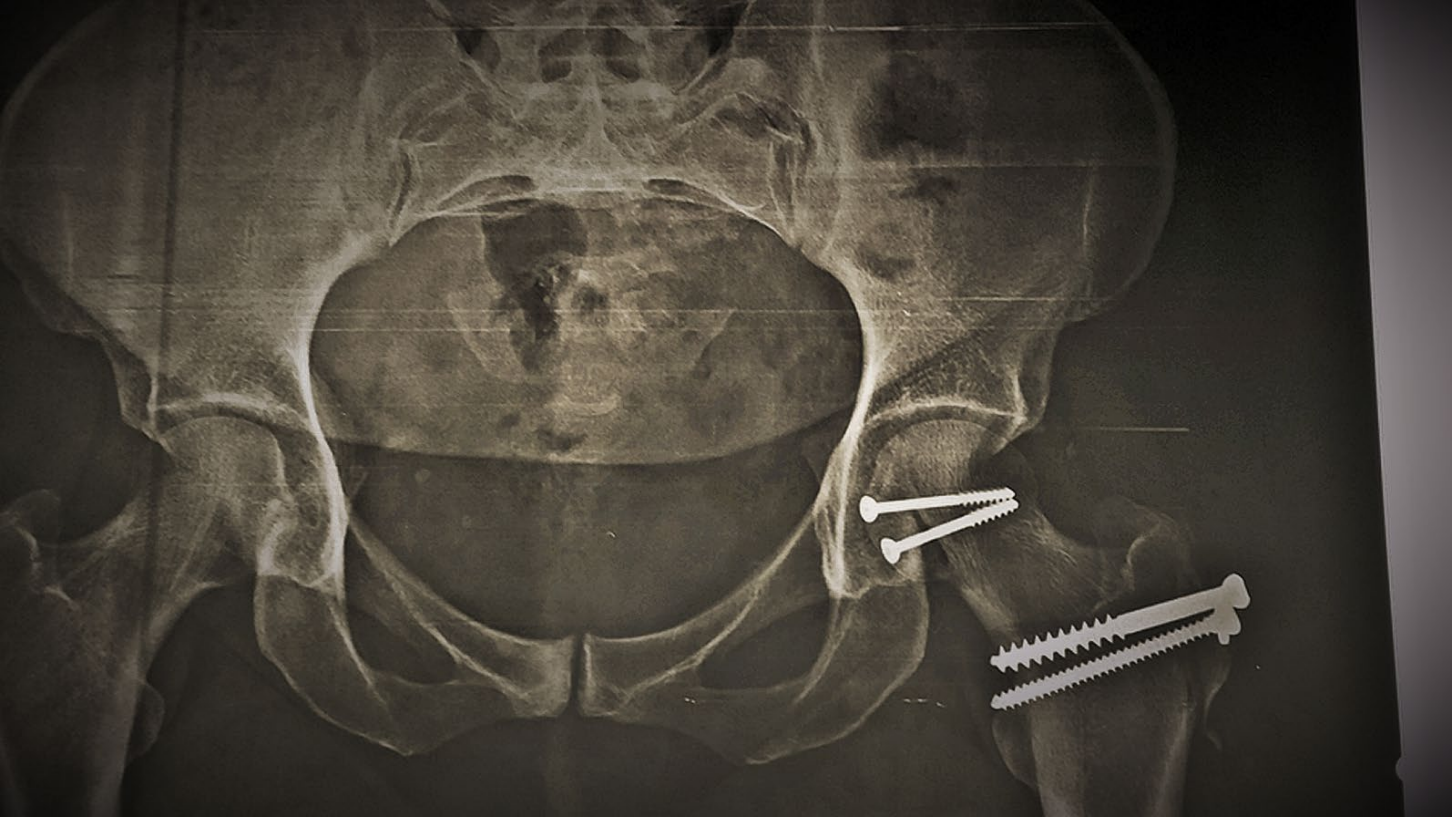
Follow-up radiograph at 1 month postoperatively

**Fig. 8:**
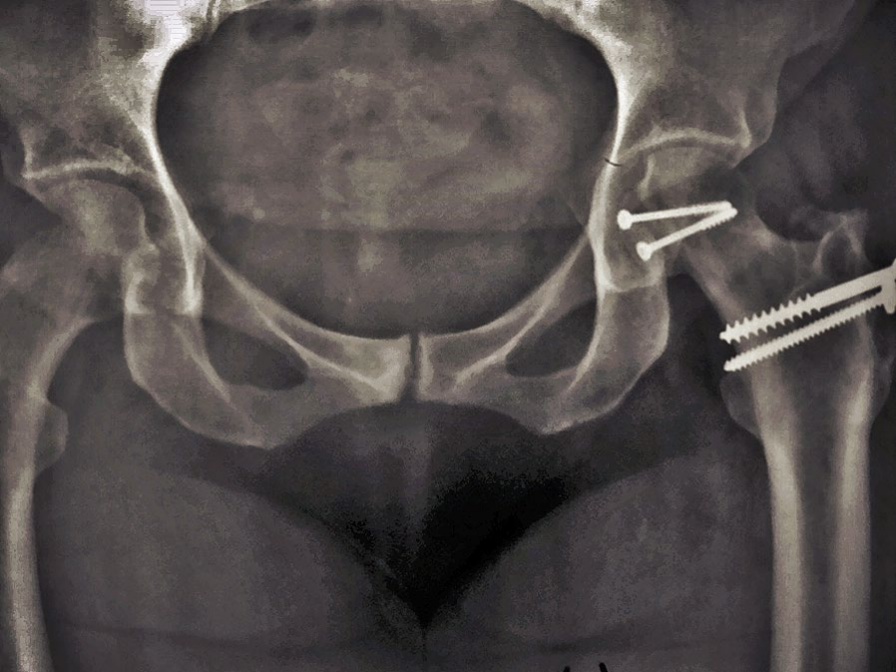
Follow-up radiograph 2 years postoperatively

**Fig. 9:**
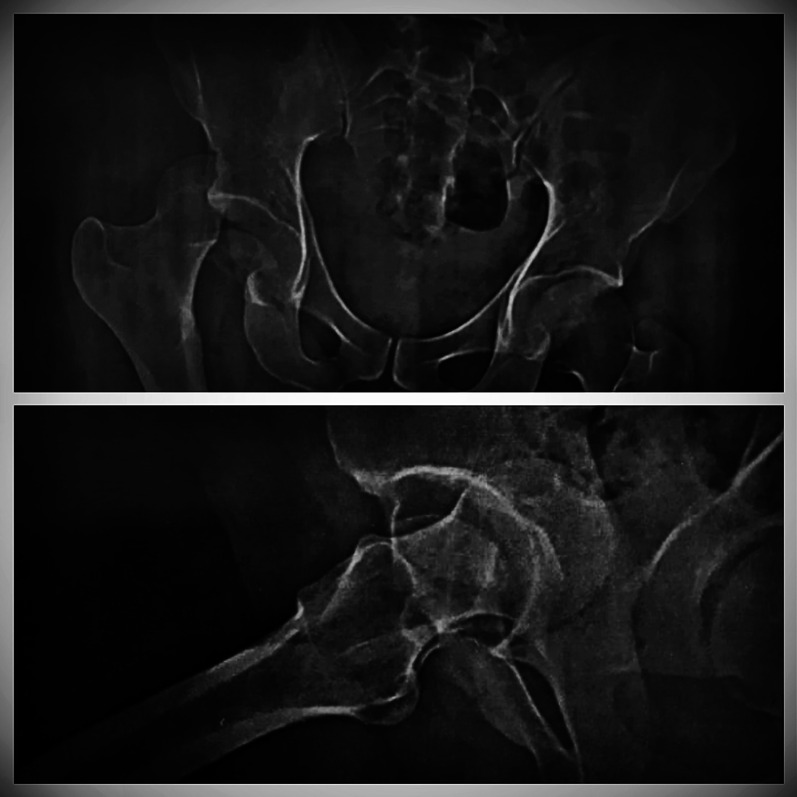
**A**,** B** Preoperative radiograph of a male 28-year-old patient with right hip dislocation and fracture of the femoral head (Pipkin type 2 fracture)

**Fig. 10:**
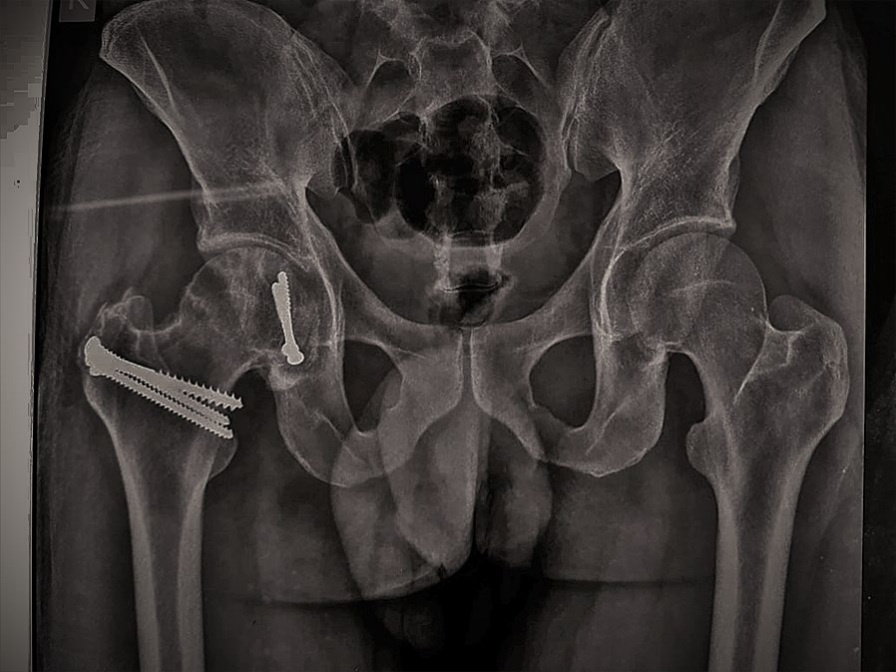
Follow-up radiograph at 12 months showing femoral head osteonecrosis

Intraoperatively, all cases had labral injury, which was debrided. None of our cases needed suture anchor repair of the labrum. Seventeen cases (94.4%) had positive bleeding test, while only one case (5.6%) had negative bleeding test. Femoral head fracture was fixed using cannulated screws 4 mm in 12 patients, while subchondral Herbert screws were used in six patients. No intraoperative or early postoperative complications were encountered throughout the study. Radiographic healing occurred at 6 weeks for both the femoral head fracture and the trochanteric osteotomy site. One case (5.6%) with Pipkin type II developed osteonecrosis at 12 months of follow-up and was scheduled for total hip replacement. The osteonecrosis was partial and was diagnosed at routine follow-up X-ray. The patient was complaining of hip pain radiating to the knee with limited internal rotation of the hip joint. The cause of osteonecrosis could not be identified. Intraoperatively, this case had a negative bleeding sign with drilling technique. Postoperative complications such as infection, wound dehiscence, heterotopic ossification, hip osteoarthritis, implant loosening or breakage, nonunion of trochanteric osteotomy, and pain or discomfort at the greater trochanter region were not experienced throughout our series Figs. 4, 5, 6, 7, 8, 9, 10. All patients returned to their normal activities except the patient who had femoral head osteonecrosis.

Radiographical evaluation according to Matta’s criteria yielded anatomic fracture reduction in 17 patients but imperfect in 1 patient. The imperfect reduction was related to the femoral head fragment as there was a comminuted friable cartilage injury at the edges of fractured femoral head fragment.

According to the Modified HHS, four Pipkin type I cases (22.2%) were rated as excellent and two (11.1%) as good, while among Pipkin type II fractures, six cases (33.3%) were rated as excellent, four (22.2%) as good, one (5.6%) as fair, and one (5.6%) as poor.

According to the modified Merle d’Aubigne and Postel score, 11 cases (61.1%) had excellent results, 5 cases (27.7%) were rated as good, one (5.6%) was fair, and one case (5.6%) had poor results.

## Discussion

Controversy still exists regarding which approach is better for treatment of femoral head fracture [22]. Surgical hip dislocation can be used for treatment of this type of fracture. It provides better visualization and the possibility to identify and eventually treat posterior wall lesions or labral tears [2, 7, 23, 24]. Surgical approaches that are used for treatment of femoral head fracture are variable. These approaches are Kocher–Langenbeck (posterior), Watson–Jones (anterolateral), Smith–Petersen (anterior), and Ludloff (medial) [7, 25].

Mostafa et al. reported that surgical hip dislocation using trochanteric osteotomy was associated with less blood loss and shorter operative time than the posterior hip approach. They concluded that surgical hip dislocation was superior to the posterior approach because of its advantages such as full exposure of the whole femoral head, direct screw fixation, and less incidence of osteonecrosis. They recommended the posterior approach for treatment of irreducible hips [2]. However, occasional complications such as fracture nonunion, migration of the osteotomized trochanter, and trochanteric bursitis may occur with trochanteric osteotomy technique [26].

Femoral head fracture is a rare injury, so few literature studies describing the technique of surgical hip dislocation in traumatic femoral head fracture exist (Table 2).

**Table 2:** Other studies concerned with surgical hip dislocation

Authors	Year	No. of cases	Indication for dislocation	Score	Follow-up period (mean, months)	Complications
Gardener et al.	2005	2 cases	Pipkin type II (2 cases)	None		–
Henle et al.	2007	12 case	Pipkin type I (1 case) Pipkin type II (3 cases) Pipkin type IV (8 cases)	Merle d’Aubigne and Postel score Thompson and Epstein score	31	Osteonecrosis ( 2 cases) Heterotopic ossification (5 cases) Reduced range of motion (4 cases)
Solberg et al.	2009	12 case	Pipkin type IV fractures	Merle d’Aubigne´and Postel score Thompson–Epstein scoring	47	Osteonecrosis (1 case) Heterotopic ossification (3 cases)
Kokubo et al.	2012	12 cases (3 cases treated with trochanteric flip osteotomy all of them of Pipkin type IV)	Pipkin type I (5 cases) Pipkin type II (2 cases) Pipkin type III (2 cases) Pipkin IV (3 cases)	Thompson and Epstein’s score	115	Osteoarthritis in one case treated later on by THA Heterotopic ossification (1 case)
Masse et al.	2014	13 case	Pipkin type I (5 cases) Pipkin type II (two cases) Pipkin type IV (6 cases)	Modified Harris hip score	77	Osteonecrosis (1 case) Heterotopic ossification (2 cases) Arthritis (1 case)
Moustafa et al.	2014	23 cases (only 12 cases treated with trochanteric flip osteotomy)	Pipkin type I ( 5 cases) Pipkin type II (18 case)	Modified Merle d’Aubigne and Postel Thompson and Epstein scores	31	Osteonecrosis (1 case) Nonunion of the trochanteric osteotomy developed in one patient
Gavaskar et al.	2015	26 case	Pipkin type I (22 case) Pipkin type II ( 4 case)	Modified Merle d’Aubigne score Oxford hip score	36	No osteonecrosis Heterotopic ossification (5 cases) Deep infection (1 case) Painful trochanteric bursitis (1 case) which relieved after screw removal
Stirma et al.	2018	4 cases	Pipkin type I (2 cases), Pipkin type II (1 case) Pipkin type IV (1 case)	Harris Hip Score	15	1 case progressed to deep surgical site infection. He underwent total hip arthroplasty after 1 year of follow-up later on 1 case evolved with heterotopic calcification No cases of femoral head necrosis were identified
Wang et al.	2019	12 case	Pipkin type I (4 cases), Pipkin type II (3 case) Pipkin type IV (5 cases)	The modified Merle d’Aubigne scores	35	Osteonecrosis (one case) Heterotopic ossification (4 cases)
Rana et al.	2020	6 cases	–	Harris Hip Score	10.8	Osteonecrosis (2 cases)
Khalifa et al.	2020	27 cases	Pipkin type I (6 cases), Pipkin type II (13 case) Pipkin type IV (8 case)	Modified Harris hip Modified Merle d’Aubigne and Postel scores	48	Osteonecrosis (2 cases) Heterotopic ossification (5 cases) Hip arthritis (1 case)
Engel et al.	2021	7 cases	Pipkin type IV (7 case)	None	894.57 ± 496.16 days	Osteonecrosis (2 cases) Heterotopic ossification (1 case) Hip arthritis (6 cases (4 converted to THA)) Deep infection (one case)
Current study	2022	18 case	Pipkin type I (6 cases), Pipkin type II (12 cases)	Modified Harris Hip and Modified Merle d’Aubigne and Postel scores	40	Osteonecrosis (1 case)

Our findings are similar to other studies in the literature. We had only one case of osteonecrosis [2, 4, 22]. Some authors reported high incidence of osteonecrosis, but it was mostly related to Pipkin type (type IV) femoral head fracture [5, 27]. Solberg reported one case of osteonecrosis among 12 patients with Pipkin type IV fracture [6]. Unlike other studies [3, 5, 6, 14, 22], complications such as osteoarthritis hip, infection, and heterotopic ossification were not encountered in in the current study.

Fixation methods for femoral head fracture are diverse. Some authors report the use of Herbert screw [2, 5, 6, 14, 22, 28], partial cancellous screw [2, 14, 22], headless screw [3, 6], and nonabsorbable screw [4]. In the current study, Herbert screw was used in 6 patients and partially cancellous screw in 12 cases. Cannulated screws provide more compression than Herbert screw [29]. We believe that compression is required in fixation of head fragment, either to enhance the healing process or to use compression to embed head fragments together and prevent their displacement.

Matta’s criteria for radiological evaluation of fracture reduction were reported in three studies [4, 14, 22] as well as the current study. Functional outcome can be evaluated either by Harris Hip Score [4, 8, 14, 28] or Merle d’Aubigne and Postel score [2, 3, 5, 6, 14, 22]. We used both Harris Hip Score and Merle d’Aubigne and Postel score for functional evaluation of our cases.

Posterior labral tear is a constant intracapsular injury in posterior wall acetabular fracture [30]. In our case series, all cases had posterior labral injury (peripheral type). We were unable to repair labral injury as it was severely torn. It was just debrided (trimming of the torn edges). None of our cases needed suture anchor repair. Some authors state that the avulsed posterior root of the labrum (osseus avulsion) is an indication for repair. It may disrupt the joint sealing function of normal labrum and induce hip joint instability [30]. Solberg et al. reported labral tears at the superior acetabulum rim in all cases [6]. Gavaskar et al. reported labral tears in 15 patients (53.5%). Tears were located in the posterosuperior region. They reported that labral tears can be safely accessed and repaired through surgical hip dislocation approach [3]. Masse et al. reported labral tears in four patients in their case series [4].

The incidence of osteonecrosis in traumatic hip dislocations is reported to range from 8% to 24% in literature [2–4, 11, 12, 31]. The main cause of osteonecrosis of the femoral head is injury to its blood supply. This may be caused initially during the traumatic hip dislocation, or during the surgical procedure.

It is difficult to differentiate the cause responsible for this injury, but Pipkin type III or IV is an indicator of high injury trauma, which may be the primary cause of osteonecrosis, so intraoperative assessment of the femoral head vascularity using the drill technique is an essential step during the surgical procedure. Moreover, the time elapsed between hip dislocation insults and femoral head reduction is key for good outcomes and to avoid osteonecrosis [3, 32, 33].

The mean incidence of osteonecrosis in trochanteric flip osteotomy is reported to be 12.5%, with 16.4% in posterior hip approach versus 7.9% in anterior approach. The anterior hip approach was associated with the lowest incidence of osteonecrosis but was associated with high incidence of heterotopic ossification [34]. Surgical hip dislocation provides better visualization and the possibility to identify and eventually treat posterior wall lesions or labral tear.

In our study, closed reduction of hip dislocation within 6 h was done for all cases, and the average time to operation was 5 days. This may explain the low incidence of osteonecrosis [one case (5.5%)]. Also, the rigorous execution of the approach keeping in mind the course of the medial femoral circumflex artery lowered the incidence of osteonecrosis and other complications.

The drilling test (bleeding sign) is one of the reliable tests to predict the viability of the femoral head in patients who undergo surgical hip dislocation [35, 36]. In a study to predict femoral head vitality during surgical hip dislocation, Aprato et al. reported that the sensitivity of the drilling test was 97%, while its specificity was 83% [35]. The test can be performed intraoperatively to determine femoral head viability. Negative drilling test means impaired head vascularity and high incidence of osteonecrosis.

In this study, intraoperative assessment of the femoral head revealed one case with negative drilling test, and this patient later presented with femoral head osteonecrosis. The intraoperative drilling test can guide the surgeon to change his surgical plan from just fixation to total hip arthroplasty after patient consent.

Despite the fact that anatomic fracture reduction was obtained in most cases (17 case), the functional outcome is different, grading from excellent to poor. This can be explained by many factors such as trauma insult to the femoral head and its cartilage, traumatic injury to the surrounding soft tissues and muscles, patient variation (age, sex, and race), and their response to rehabilitation program, pain tolerance, and patient satisfaction. According to the current study, we believe that there is no relation between fracture reduction and functional outcome. The functional outcome is affected by many factors. Fracture reduction, even if it is anatomical, does not mean that an excellent functional outcome will be obtained. This point requires further study.

One limitation of this study is the small number of cases, making statistical analysis difficult to perform, although the number of cases can be considered excellent when compared with other studies in literature. Moreover, this shortage is considered to be an obstacle to comparison between surgical hip dislocation and other techniques. Another limitation of this study is the short follow-up period. Osteonecrosis and osteoarthritis course is unexpected and unpredictable.

## Conclusion

Open reduction and internal fixation of femoral head fracture using surgical hip dislocation through Ganz approach is a viable treatment option and provides satisfactory results with low complication rates.

## Data Availability

All data and material are available.
